# Comments on: “The relationship between parental mental health and early childhood caries: a meta-analysis and systematic review”

**DOI:** 10.1038/s41405-026-00449-6

**Published:** 2026-05-27

**Authors:** K. Julia Hurry, Tim Newton, Janet Davies, Vanessa Muirhead

**Affiliations:** 1 Institute of Dentistry, Faculty of Medicine and Dentistry, Queen Mary University of London, London, UK; 2 Faculty of Dentistry, Oral & Craniofacial Sciences, King’s College London, London, UK

**Keywords:** Paediatric dentistry, Oral-health-related quality of life

We read with interest the systematic review and meta-analysis by Ma et al. [1] that explores the relationship between parental mental health and early childhood caries (ECC) [1]. Reporting a pooled odds ratio of 1.54 for ECC prevalence among children of parents with mental health conditions, the authors conclude that “vigilant monitoring and proactive management of parental mental health may be a key strategy for reducing ECC risk”. However, we suggest this conclusion goes beyond what the evidence can reliably support.

A key limitation is the observational nature of the included studies. Of the 12 studies, nine were cross-sectional and only three used cohort studies, limiting causal inference and raising the possibility of reverse causation [1]. In addition, adjustment for confounding was inconsistent: only four of the nine cross-sectional studies and one of the three cohort studies controlled for key confounders [1]. This is important because ECC is strongly associated with socioeconomic status, diet, and access to care [2, 3]. Without adequate adjustment, the reported association may reflect shared underlying disadvantage rather than a direct effect of parental mental health.

Evidence from the wider literature supports this interpretation. Socioeconomic disadvantage directly impacts ECC, while psychosocial factors, such as parental resilience, influence indirectly through oral health behaviours, such as diet, oral hygiene and dental attendance [4]. Similarly, longitudinal studies suggest that psychosocial risk influences ECC largely through these intermediary pathways rather than acting independently [5]. This highlights the importance of distinguishing between confounders and mediators, an analytical step not clearly addressed in this review.

Although Ma et al. [1] briefly refer to possible behavioural and microbial mechanisms, these are not explored in a structured way [1]. The absence of any formal discussion of mediation limits how the findings can be interpreted. For example, parental depression might affect a child’s oral health through reduced toothbrushing supervision, changes in feeding habits, or lower dental attendance. Equally, both parental mental health and ECC may be shaped by upstream social and economic adversity [6]. Without examining these pathways, this review risks confusing correlation with causation.

Issues of framing are also important. As discussed by Allchin and Isobel (2024), research in this area has often adopted a “vulnerability and risk” narrative that, while useful for policy advocacy, can inadvertently individualise systemic problems and portray families through a deficit-oriented and victim-blaming lens. Applying this perspective to Ma et al. [1], who suggest that monitoring and managing parental mental health could reduce ECC, may inadvertently reinforce this narrative [1]. It positions parental psychological distress as a primary, modifiable driver of children’s oral disease, and risks overlooking the wider structural factors that shape both mental health and oral health outcomes.

The substantial heterogeneity reported in the meta-analysis (*I*² = 74.7%) further limits confidence in the pooled estimates [1]. Differences in how mental health and ECC were measured, along with variations in study populations, make it difficult to draw consistent conclusions. The lack of clear association for some exposure, such as parental stress in adjusted analyses, also suggests the relationship is not consistent across different aspects of mental health.

Taken together, the recommendation to focus on parental mental health as a strategy for preventing ECC is an oversimplification of a multifactorial disease. It may also shift responsibility towards parents (particularly mothers who were the primary focus of most included studies) and could contribute to stigma, particularly given the reliance on self-reported mental health measures, which are susceptible to contextual and reporting biases [1].

A more cautious interpretation would place parental mental health within a broader framework of ECC risk, where psychosocial factors interact with social and behavioural determinants rather than acting alone [3, 7]. Future reviews should consider longitudinal designs, consistently adjust for confounders and carefully examine potential mediator pathways. This would help clarify whether parental mental health is a causal factor, a mediator, or simply a marker of wider vulnerability.

In conclusion, while this review brings together an important body of evidence, its conclusions should be interpreted with caution. We as researchers can play an essential role to reduce stigma by avoiding overstating findings and conflating association with causation in both our research and public health messaging.
